# Csf1: A Putative Lipid Transport Protein Required for Homeoviscous Adaptation of the Lipidome

**DOI:** 10.1177/25152564221101974

**Published:** 2022-06-28

**Authors:** Arun T. John Peter, Ngaam J. Cheung, Benoît Kornmann

**Affiliations:** 1 Department of Biology, 30474 University of Fribourg, Switzerland; 2 Department of Biochemistry, 98957 University of Oxford, UK

**Keywords:** lipid transport, chorein-N motif, lipid remodeling, membrane contact sites, homeoviscous adaptation

## Abstract

The non-vesicular transport of lipids between organelles mediated by lipid transport proteins (LTPs) is a key determinant of organelle biogenesis and function. Despite performing a vital function in organelle homeostasis, none of the LTP-encoding genes identified so far are truly essential, even in the simple genome of yeast, suggesting widespread redundancy. In line with this fact, it has been found that a number of LTPs have overlapping functions, making it challenging to assign unique roles for an individual LTP in lipid distribution. In our genetic screens under stringent conditions in which the distinct function of an LTP might become essential, we stumbled upon Csf1, a highly conserved protein with a Chorein-N motif found in other lipid transporters and unraveled a new function for Csf1 in lipid remodeling and homeoviscous adaptation of the lipidome. Here, we further speculate on the potential mechanisms of how the putative function of Csf1 in lipid transport could be intimately connected to its role in lipid remodeling across organelles.

Lipid transport is a critical cellular process that underlies the biogenesis and distinct functions of organelles. The distribution of lipids from their site of synthesis, mainly the endoplasmic reticulum (ER), to a destination organelle can be mediated by vesicles, or by lipid transport proteins (LTPs) in a non-vesicular fashion. LTPs, which operate at sites of close contact between organelle membranes, are thought to mediate the bulk of lipid transport by solubilizing lipids from a donor membrane and transferring them to an acceptor membrane (Wong et al., 2019). There are ∼40 LTPs identified so far in yeast. Despite their functional importance in lipid transport, surprisingly, none of the LTP-encoding genes are essential, suggesting that LTPs may have overlapping functions. Indeed, ER-mitochondria encounter structures (ERMES), a complex of three LTPs, and the Chorein-N motif protein Vps13 function redundantly to distribute lipids between ER and mitochondria (John Peter et al., 2017, 2021; Kumar et al., 2018; Lang et al., 2015). At the same time, the fact that there is a selection pressure to maintain the seemingly redundant genes encoding different LTPs suggests non-redundant functions, which might be essential in a specialized physiological context. Lipid adaptation to physiological contexts is well documented (Ernst et al., 2016). One of the best examples is homeoviscous adaptation, an adaptive response by which cells adapt their lipidome to environmental temperatures. For instance, in the cold, cells usually remodel their membranes to maintain a constant membrane viscosity. One mode of remodeling involves altering the ratio between phosphatidylethanolamine (PE) and phosphatidylinositol (PI). While PE increases membrane fluidity owing to its curvature-promoting smaller headgroup, PI has the opposite effect due its ability to form hydrogen bonds between its head group moieties (Klose et al., 2012). Another mode of membrane adaptation entails incorporation of lipids with shorter and more unsaturated fatty acid chains. In yeast, a central player of homeoviscous adaptation is the stearyl-CoA desaturase Ole1, which is transcriptionally induced in the cold through a well-documented sensing pathway (Covino et al., 2016; Hoppe et al., 2000). Unsaturated products of Ole1 are then incorporated in membrane lipids in the ER. How ER-synthesized unsaturated lipids are subsequently distributed in other organelles, however, is unexplored.

In our recent study (John Peter et al., 2022), we re-engineered native lipid synthesis pathways to create stringent conditions in which survival of yeast cells is dependent on successful exchange of lipids between organelles. We targeted lipid biosynthesis enzymes to ectopic organelles to make lipid transport in and out of these organelles particularly important for cell growth. We then performed genetic screens in these artificial conditions to unbiasedly find the factors and the LTPs that underlie viability.

Of the wide range of functionally different genes we identified, we focused on Csf1, a single-pass ER membrane protein conserved in fungi and animals. Csf1 bears a characteristic Chorein-N motif found in lipid transport proteins like Vps13 and Atg2 (Cai et al., 2022; Kumar et al., 2018; Li et al., 2020; Osawa et al., 2019; Valverde et al., 2019). *CSF1* became essential in an artificial condition in which phosphatidylethanolamine (PE) was synthesized exclusively in the mitochondrial inner membrane and phosphatidylcholine (PC) in peroxisomes. While Csf1's absence did not cause a defect in PE and PC biosynthesis even in these artificial conditions, a cue to its role came from a previous finding that it is required for growth in cold temperature (Tokai et al., 2000). Indeed, it turned out that the artificial condition on its own caused cold sensitivity, explaining the synthetic lethality with *csf1*, and suggesting that both conditions yielded a lipidome ill-adapted to cold. Specifically, we found that remodeling of the lipidome from saturated to mono- and mono- to di-unsaturated species upon cold exposure was compromised by the loss of Csf1.

What is the possible link between the potential role of Csf1 in lipid transport and the observed function in lipid remodeling? We and others have observed that Csf1 has a punctate localization along the ER, with those dots potentially in contact with the plasma membrane (PM) (Castro et al., 2021; John Peter et al., 2022; Toulmay et al., 2022). One possibility is that Csf1 imports lipids from the PM and provides them as substrates to the ER-localized lipases to generate free fatty acids, which can then be activated as fatty-acyl-CoA and provided as substrate for the desaturase Ole1 to make unsaturated lipids (Figure 1). Another non-exclusive possibility is that newly generated unsaturated lipids use Csf1 to return to the PM. In addition, mitochondria might serve as a lipid source and/or destination as a subset of Csf1 puncta co-localize with the ERMES subunit Mmm1 (Toulmay et al., 2022). As one end of Csf1 is anchored to the ER via its transmembrane domain, it is possible that, like for Vps13 (Bean et al., 2018; John Peter et al., 2017), the other end of the protein can be dynamically recruited to PM or mitochondria via adapter proteins. Speculatively, Csf1 might selectively import or export saturated or unsaturated lipid species to the ER, potentially explaining its unique role in homeoviscous adaptation. We recently showed that redundant LTPs might in fact have some preference for lipids with a specific fatty acid configuration (John Peter et al., 2021). Alternatively, Csf1 might be able to channel lipids directly to ER-localized lipases or transport lipids in a certain direction that is critical for adaptation to cold.

**Figure 1.**
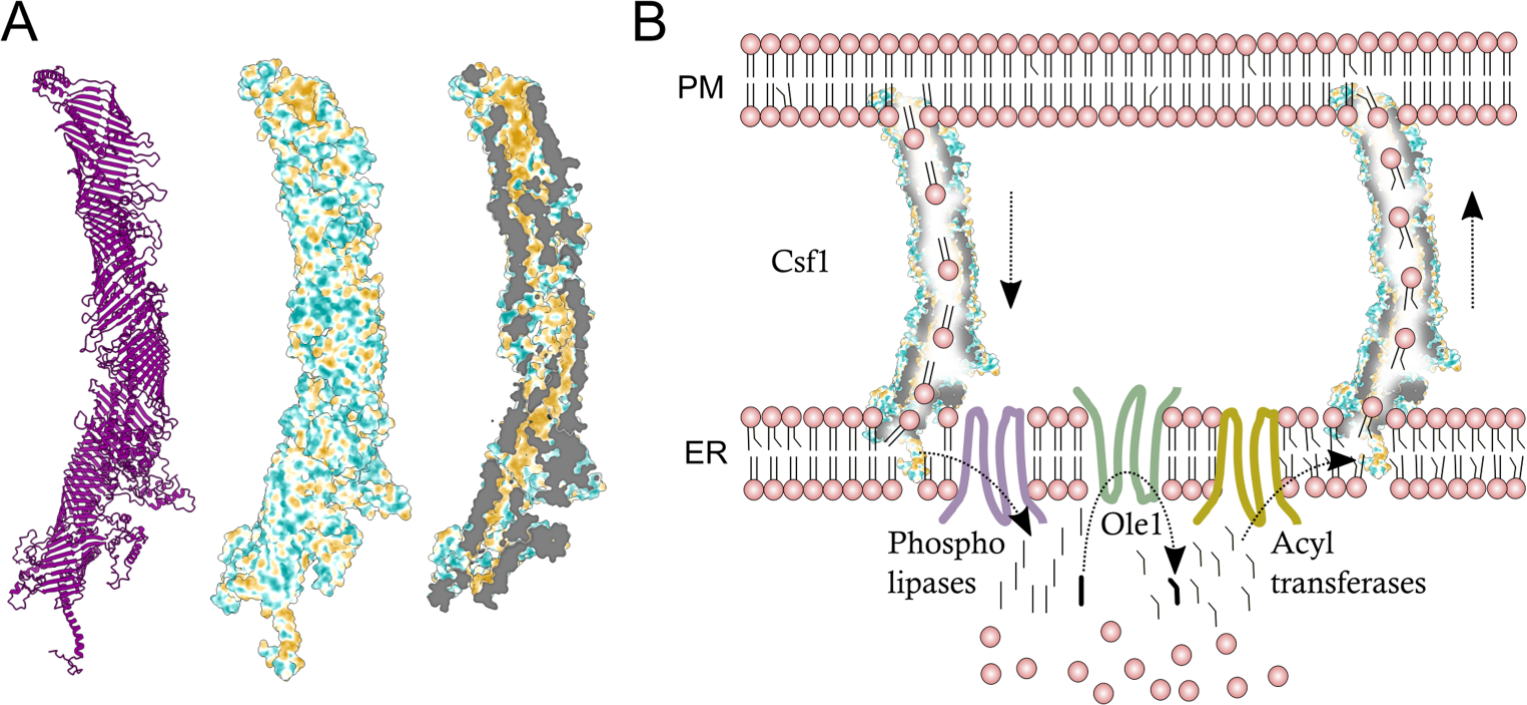
A: Predicted structure of yeast Csf1 with the cross-sectional view (right) revealing hydrophobic residues (in yellow) along the groove. The structure was predicted using in-house scripts based on *Leri* (Cheung et al., 2021), with constraints of torsional angles and residue-distance map. B: A hypothetical model that proposes a role for Csf1 in lipid import and/or export to/from the ER and facilitating the production of unsaturated lipids by the ER-localized desaturase Ole1.

The recent elucidations and predictions of 3D structures for several Chorein-N motif-containing proteins like Vps13 and Atg2 has revealed a hydrophobic groove fold that can facilitate bulk transport of lipid molecules between membranes (Cai et al., 2022; Kumar et al., 2018; Li et al., 2020; Valverde et al., 2019). As cold exposure likely requires a quick adaptative response, at timescales much faster than the transcriptional upregulation of Ole1 can achieve, it is conceivable that the requirement of Csf1 is due to its lipid transport activity for downstream remodeling of the lipidome.

With multiple observations pointing to a very likely role for Csf1 in lipid transport, our discovery of its requirement in homeoviscous adaptation has opened new avenues to understand the molecular principles of how cells coordinate lipid transport and lipid remodeling. If indeed Csf1 turns out to be an authentic LTP, understanding its regulation in vivo during normal and cold temperatures, the functional significance of its interaction with mitochondria and PM, the selective nature of the hydrophobic groove as well as the directionality of lipid movement will significantly enhance our knowledge on lipid transport mechanisms at membrane contact sites.

## References

[bibr1-25152564221101974] Bean B. D. M. Dziurdzik S. K. Kolehmainen K. L. Fowler C. M. S. Kwong W. K. Grad L. I. Davey M. Schluter C. Conibear E . (2018). Competitive organelle-specific adaptors recruit Vps13 to membrane contact sites. Journal of Cell Biology, 217(10), 3593–3607. 10.1083/jcb.201804111. 30018089 PMC6168272

[bibr2-25152564221101974] Cai S. Wu Y. Guillen-Samander A. Hancock-Cerutti W. Liu J. Camilli P. D . (2022). In situ architecture of the lipid transport protein VPS13C at ER-lysosomes membrane contacts. bioRxiv. 10.1101/2022.03.08.482579. PMC9303930 35858323

[bibr3-25152564221101974] Castro I. G. Shortill S. P. Dziurdzik S. K. Cadou A. Ganesan S. Fenech E. J. Meyer H. Fadel A. David Y. Davey M. Mattes C. Valenti R. Ernst R. Zaremberg V. Levine T. P. Stefan C. J. Conibear E. Schuldiner M . (2021). Systematic analysis of membrane contact sites in saccharomyces cerevisiae uncovers modulators of cellular lipid distribution. bioRxiv. 10.1101/2021.10.17.464712. PMC9648973 36354737

[bibr4-25152564221101974] Cheung N. J. Peter A. T. J. Kornmann B . (2021). Leri: A web-server for identifying protein functional networks from evolutionary couplings. Computational and Structural Biotechnology Journal, 19, 3556–3563. 10.1016/j.csbj.2021.06.002. 34257835 PMC8239741

[bibr5-25152564221101974] Covino R. Ballweg S. Stordeur C. Michaelis J. B. Puth K. Wernig F. Bahrami A. Ernst A. M. Hummer G. Ernst R . (2016). A eukaryotic sensor for membrane lipid saturation. Molecular Cell, 63(1), 49–59. 10.1016/j.molcel.2016.05.015. 27320200

[bibr6-25152564221101974] Ernst R. Ejsing C. S. Antonny B . (2016). Homeoviscous adaptation and the regulation of membrane lipids. Journal of Molecular Biology, 428(24), 4776–4791. 10.1016/j.jmb.2016.08.013. 27534816

[bibr7-25152564221101974] Hoppe T. Matuschewski K. Rape M. Schlenker S. Ulrich H. D. Jentsch S . (2000). Activation of a membrane-bound transcription factor by regulated ubiquitin/proteasome-dependent processing. Cell, 102(5), 577–586. 10.1016/s0092-8674(00)00080-5. 11007476

[bibr8-25152564221101974] John Peter A. T. Herrmann B. Antunes D. Rapaport D. Dimmer K. S. Kornmann B. (2017). Vps13-Mcp1 interact at vacuole–mitochondria interfaces and bypass ER–mitochondria contact sites. Journal of Cell Biology, 216(10), 3219–3229. 10.1083/jcb.201610055. 28864540 PMC5626531

[bibr9-25152564221101974] John Peter A. T. Peter M. Kornmann B. (2021). Interorganelle lipid flux revealed by enzymatic mass tagging in vivo. bioRxiv. 10.1101/2021.08.27.457935.

[bibr10-25152564221101974] John Peter A. T. van Schie S. N. S. Cheung N. J. Michel A. H. Peter M. Kornmann B. (2022). Rewiring phospholipid biosynthesis reveals resilience to membrane perturbations and uncovers regulators of lipid homeostasis. EMBO Journal, 41(7), e109998. 10.15252/embj.2021109998. 35188676 PMC8982615

[bibr11-25152564221101974] Klose C. Surma M. A. Gerl M. J. Meyenhofer F. Shevchenko A. Simons K . (2012). Flexibility of a eukaryotic lipidome – insights from yeast lipidomics. Plos One, 7(4), e35063. 10.1371/journal.pone.0035063. 22529973 PMC3329542

[bibr12-25152564221101974] Kumar N. Leonzino M. Hancock-Cerutti W. Horenkamp F. A. Li P. Lees J. A. Wheeler H. Reinisch K. M. Camilli P. D . (2018). VPS13A And VPS13C are lipid transport proteins differentially localized at ER contact sites. Journal of Cell Biology, 217(10), 3625–3639. 10.1083/jcb.201807019. 30093493 PMC6168267

[bibr13-25152564221101974] Lang A. B. Peter A. T. J. Walter P. Kornmann B . (2015). ER–Mitochondrial junctions can be bypassed by dominant mutations in the endosomal protein Vps13. Journal of Cell Biology, 210(6), 883–890. 10.1083/jcb.201502105 26370498 PMC4576869

[bibr14-25152564221101974] Li P. Lees J. A. Lusk C. P. Reinisch K. M . (2020). Cryo-EM reconstruction of a VPS13 fragment reveals a long groove to channel lipids between membranes. Journal of Cell Biology, 219(5), e202001161. 10.1083/jcb.202001161. 32182622 PMC7199853

[bibr15-25152564221101974] Osawa T. Kotani T. Kawaoka T. Hirata E. Suzuki K. Nakatogawa H. Ohsumi Y. Noda N. N . (2019). Atg2 mediates direct lipid transfer between membranes for autophagosome formation. Nature Structural & Molecular Biology, 26(4), 281–288. 10.1038/s41594-019-0203-4. 30911189

[bibr16-25152564221101974] Tokai M. Kawasaki H. Kikuchi Y. Ouchi K. (2000). Cloning and characterization of the CSF1 gene of Saccharomyces cerevisiae, which is required for nutrient uptake at low temperature. Journal of Bacteriology, 182(10), 2865–2868. 10781556 10.1128/jb.182.10.2865-2868.2000 PMC101996

[bibr17-25152564221101974] Toulmay A. Whittle F. B. Yang J. Bai X. Diarra J. Banerjee S. Levine T. P. Golden A. Prinz W. A . (2022). Vps13-like proteins provide phosphatidylethanolamine for GPI anchor synthesis in the ER. Journal of Cell Biology, 221(3), e202111095. 10.1083/jcb.202111095. 35015055 PMC8757616

[bibr18-25152564221101974] Valverde D. P. Yu S. Boggavarapu V. Kumar N. Lees J. A. Walz T. Reinisch K. M. Melia T.J . (2019). ATG2 Transports lipids to promote autophagosome biogenesis. Journal of Cell Biology, 218(6), 1787–1798. 10.1083/jcb.201811139. 30952800 PMC6548141

[bibr19-25152564221101974] Wong L. H. Gatta A. T. Levine T. P . (2019). Lipid transfer proteins: The lipid commute via shuttles, bridges and tubes. Nature Reviews Molecular Cell Biology, 20(2), 85–101. 10.1038/s41580-018-0071-5. 30337668

